# Genome-wide screening identifies a *KCNIP1* copy number variant as a genetic predictor for atrial fibrillation

**DOI:** 10.1038/ncomms10190

**Published:** 2016-02-02

**Authors:** Chia-Ti Tsai, Chia-Shan Hsieh, Sheng-Nan Chang, Eric Y. Chuang, Kwo-Chang Ueng, Chin-Feng Tsai, Tsung-Hsien Lin, Cho-Kai Wu, Jen-Kuang Lee, Lian-Yu Lin, Yi-Chih Wang, Chih-Chieh Yu, Ling-Ping Lai, Chuen-Den Tseng, Juey-Jen Hwang, Fu-Tien Chiang, Jiunn-Lee Lin

**Affiliations:** 1 Division of Cardiology, Department of Internal Medicine, National Taiwan University College of Medicine and Hospital, No. 7, Chung-Shan South Road, Taipei 100, Taiwan; 2 Graduate Institute of Clinical Medicine, College of Medicine, National Taiwan University, No. 7, Chung-Shan South Road, Taipei 100, Taiwan; 3 Department of Internal Medicine, National Taiwan University Hospital Yun-Lin Branch, No. 579, Sec. 2, Yunlin Road, Douliou City, Yunlin County 640, Taiwan; 4 Department of Life Science, Genome and Systems Biology Degree Program, National Taiwan University, No. 1, Sec. 4, Rutherford Road, Taipei 10617, Taiwan; 5 Bioinformatics and Biostatistics Core, Center of Genomic Medicine, National Taiwan University, No. 2, Syu-jhou Road, Taipei 10055, Taiwan; 6 School of Medicine, Chung Shan Medical University, No. 110, Sec. 1, Jianguo North Road, Taichung City 40201, Taiwan; 7 Department of Medicine, Chung Shan Medical University Hospital, No. 110, Sec. 1, Jianguo North Road, Taichung City 40201, Taiwan; 8 Division of Cardiology, Department of Internal Medicine, Kaohsiung Medical University and Chung-Ho Memorial Hospital, No. 100, Shi-Chuan 1st Road, Kaohsiung City 807, Taiwan; 9 Department of Laboratory Medicine, National Taiwan University College of Medicine and Hospital, No. 7, Chung-Shan South Road, Taipei 100, Taiwan

## Abstract

Atrial fibrillation (AF) is the most common sustained cardiac arrhythmia. Previous genome-wide association studies had identified single-nucleotide polymorphisms in several genomic regions to be associated with AF. In human genome, copy number variations (CNVs) are known to contribute to disease susceptibility. Using a genome-wide multistage approach to identify AF susceptibility CNVs, we here show a common 4,470-bp diallelic CNV in the first intron of potassium interacting channel 1 gene (*KCNIP1)* is strongly associated with AF in Taiwanese populations (odds ratio=2.27 for insertion allele; *P*=6.23 × 10^−24^). *KCNIP1* insertion is associated with higher *KCNIP1* mRNA expression. *KCNIP1*-encoded protein potassium interacting channel 1 (KCHIP1) is physically associated with potassium Kv channels and modulates atrial transient outward current in cardiac myocytes. Overexpression of *KCNIP1* results in inducible AF in zebrafish. In conclusions, a common CNV in *KCNIP1* gene is a genetic predictor of AF risk possibly pointing to a functional pathway.

## Abstract (web summary)

Tsai *et al*. here utilize a multi-stage genome-wide association study in Taiwanese population to show a copy number variation in the intron of potassium interacting channel 1 gene (KCNIP1) to be strongly associated with atrial fibrillation. The study also examines the functionality of KCNIP1 in heart electrophysiological function using cultured myocytes and zebrafish.

## Graphical abstract

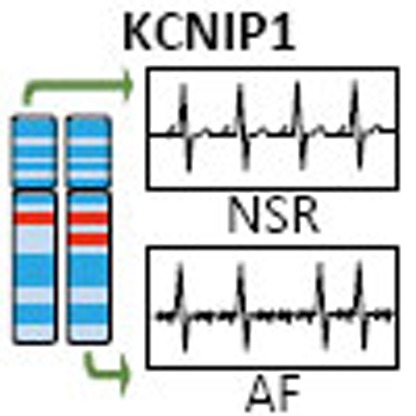

Atrial fibrillation (AF) is the most common sustained arrhythmia and a major risk factor for stroke, heart failure and cardiovascular death. In the past decade, genome-wide association studies (GWASs) have identified single-nucleotide polymorphisms (SNPs) in several genomic regions associated with AF, for example, on chromosomes 4q25 (*PITX2*), 16q22 (*ZFHX3*) and 1q21 (*KCNN3*)^1^,^2^,^3^. However, these loci do not fully explain the genetic risk for AF, suggesting that additional genetic factors or variants remain to be discovered. In the human genome, copy number variation (CNV) is a variation in the DNA sequence and can affect the expression and function of nearby and distal genes^4^, causing phenotypic differences. It has been demonstrated that signals from SNPs and CNVs have little overlap^4^. Therefore, examining the genome for both SNP and CNV variants might be an effective means of determining the genetic causes of complex phenotypes and diseases in humans.

CNV regions have been estimated to cover 5% of the human genome^5^. Inherited CNVs underlie Mendelian diseases, and some copy number (CN) variable genes are associated with rare human diseases, such as schizophrenia and autism^6^,^7^. Although some studies have shown that CNVs may also contribute to the susceptibility to common diseases^8^, their influence on phenotypic variability and disease susceptibility still remained poorly understood.

Whether CNVs may also contribute to the risk of AF has never been investigated before. Herein, we sought to explore this issue using a genome-wide approach and a three-stage study design with the attempt to minimize false positive findings yet maximize power and efficiency by examining samples with gradually increased phenotypic severity but with increasing sample size^9^. A similar approach had been used to efficiently identify the genetic susceptibility loci associated with electrocardiographic QT interval by GWAS^9^.

In addition, so far there have been many genetic studies adopting different genome-wide technologies to identify causal variants or susceptibility genes for common diseases, for example, GWAS^1^,^10^ or whole exome sequencing^11^. However, few of the identified variants or loci have been linked to disease mechanisms with translational functional studies^2^,^12^. Hence, in the present study, we identify a common diallelic insertion/deletion CNV in the Kv channel interacting protein 1 gene (*KCNIP1*) as a strong predictor of AF susceptibility and, using the zebrafish and cellular models, we also provide the possible functional mechanism to explain the genetic association. *KCNIP1*-encoded protein potassium channel interacting protein 1 (KCHIP1) may modulate atrial electrophysiology at high atrial rates.

## Results

### General CNV pattern and association with the risk of AF

Genome-wide detection of CNV was performed in the stage I discovery subjects, using the Illumina HumanOmni1-Quad BeadChip (1,014,075 SNPs) to obtain signal and allelic intensities and to generate CNV calls. After quality control filtering (removal of markers or subjects with call rate<99%), there was a total of 7,210 CNV calls. The CNV calls spanned between 1 and 2,288 SNP markers, with an average of 43 SNPs per CNV region and an average CNV region size of 119 kb.

Although the small stage I sample size, we still identified several CNV regions that were associated with AF (Table 1), probably because of the significant phenotype contrast between cases (extreme phenotypes) and controls. All of the significant CNVs were small CNVs (<500 kb) and, thus, there was no enrichment of large CNVs among the cases relative to controls as observed in Mendelian diseases. Because of the smaller case number in the stage I GWAS sample (because of very low prevalence of extreme AF cases), false positive results were expected by chance, and further follow-up was critical.

We then replicated the significant CNVs from the stage I discovery sample in the stage II replication sample with another 105 patients with symptomatic AF and 422 normal sinus rhythm (NSR) healthy controls. Interestingly, we found that the CNV region in an ionic channel/subunit gene, the Kv channel interacting protein 1 gene (*KCNIP1*), remained significant in the stage II sample. We found a short (∼4,470 bp) common insertion/deletion diallelic CN polymorphism in the first intron of human *KCNIP1* gene that was associated with the susceptibility to AF. The insertion allele of the diallelic CNV was associated with an increased risk of AF, and the deletion allele was protective from AF (harbouring insertion allele was associated with AF, *P*=1.8 × 10^−5^). This diallelic CNV has also been published recently to be associated with the risk of type 2 diabetes^13^. Furthermore, it has also been published in the Database of Genomic Variants (http://projects.tcag.ca/variation) and the 1,000 Genomes (http://www.1000genomes.org)(Supplementary Fig. 1).

CNVs may be linked to their neighbouring SNPs. We showed the regional plot around this *KCNIP1* CNV region (Chr5:169,800,000–170,300,000) (Supplementary Fig. 2). In this region, only rs11742875, which is ∼100 kb far from the CNV, was associated with AF based on the preset stage I threshold of 0.001 (*P*=1.05 × 10^−4^). However, the density of SNP in the chip is sparse, and there may be other better SNP markers around the CNV^14^. Based on the 1000 Genomes data, this CNV (MERGED_DEL_2_33224) is in very strong linkage disequilibrium (*r*^2^>0.95) with three SNPs (rs1541665, rs2032863 and rs1363713). SNP rs1541665 was among the SNP list of our Illumina HumanOmni1-Quad BeadChip (Supplementary Fig. 2), but not rs2032863 and rs1363713. However, the *r*^2^ between rs1541665 and the CNV was not high (0.48) in our Taiwanese population, and therefore rs1541665 was not significantly associated with AF trait (*P*=0.093) (Supplementary Fig. 2). We also genotyped rs2032863 and rs1363713. SNP rs2032863 was in complete linkage disequilibrium with rs1363713 and therefore provided no additional information. The *r*^2^ between rs1363713 and the CNV was modestly high (0.78) in our Taiwanese population, but not as strong as found in other populations (>0.95). Therefore, the pattern of linkage disequilibrium between genetic marks may be variable in different ethnic populations^15^. Nevertheless, the association of rs1363713 with AF trait was highly significant (*P*=3.34 × 10^−5^), but not as significant as the CNV. In conclusions, this CNV might be only partially tagged by surrounding SNPs, and therefore, genotyping of this common CNV was still necessary for the large-scale stage III association study.

Accordingly, we further validated the association of this common CNV with AF in the large stage III population. The distribution of *KCNIP1* insertion/deletion genotypes was also in Hardy–Weinberg equilibrium in the stage III sample (Table 2). Similarly, the insertion allele was associated with an increased risk of AF (insertion allele frequency 0.36 versus 0.23, *P*=5.38 × 10^−23^). The association became even more significant because of the large case number. Harbouring at least one insertion allele was associated with a significantly higher risk of AF (odds ratio 2.12, 95% confidence interval 1.54–2.94, *P*=4.19 × 10^−22^).

### Validation in different geographic areas

In stage III, we also genotyped this common insertion/deletion polymorphism in 821 subjects (275 AF cases and 546 NSR controls) in different geographic areas (Middle and Southern Taiwan). Power estimation revealed that we had >95% power to replicate the association for an odds ratio of 2.0 at an alpha level of 0.05. The distribution of *KCNIP1* insertion/deletion genotypes was also in Hardy–Weinberg equilibrium (Table 2). Again, we observed a significantly association between insertion allele and AF (autosomal dominant odds ratio 2.38, 95% confidence interval 1.75–3.23, *P*=1.08 × 10^−8^). Given this strong genetic association between *KCNIP1* gene and AF, we tried to investigate the possible underlying functional mechanism.

### Expression of *KCNIP1* in the mammalian atrium

*KCNIP* genes encode the potassium channel interacting proteins (KCHIPs). KCHIPs are small cytosolic, calcium-binding proteins that were initially identified as subunits for the voltage-gated A-type potassium current in neuronal tissue and the transient outward potassium current in cardiac tissue^16^. The pore-forming protein complex governing these potassium currents is the Kv4 protein. KCHIPs modulate the kinetics and current amplitudes of these Kv4 currents^16^. Four *KCNIP* genes have been identified in humans and mice, encoding KCHIP1-4 (refs 16, 17). KCHIP1 is mainly expressed in neuronal tissues^18^, whereas KCHIP2 is mainly expressed in cardiac tissues^16^,^17^. However, whether KCHIP1 is expressed in the human atrium has never been investigated before.

**Figure 1:**
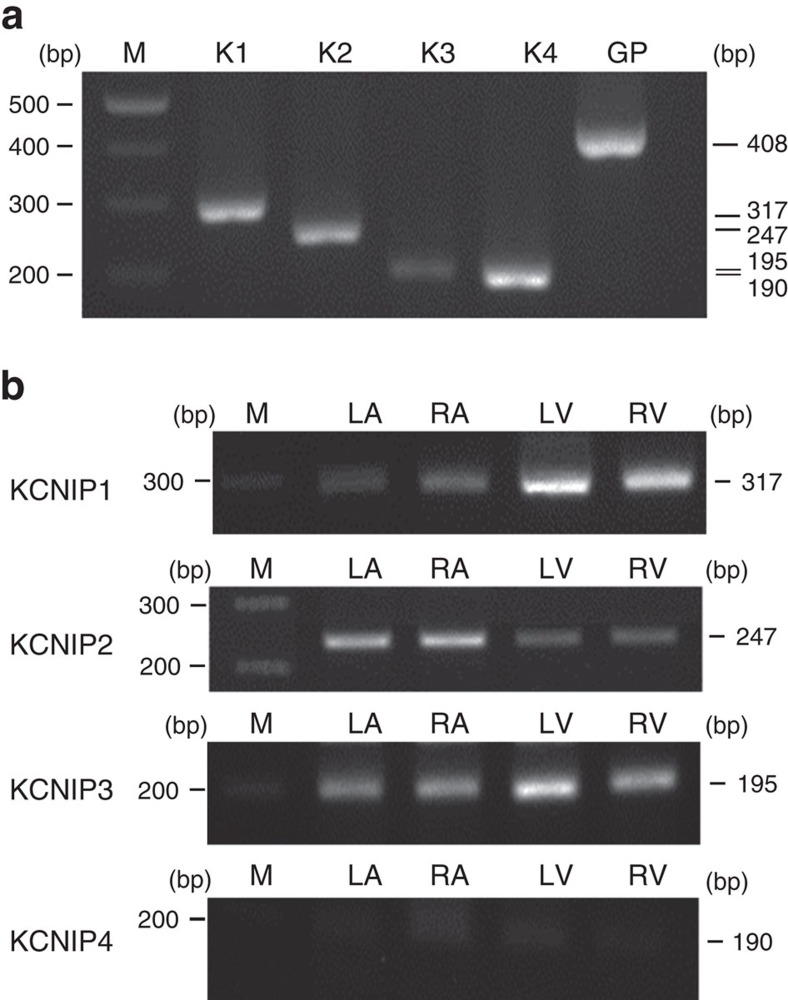
Basal expressions of *KCNIP*s in the mammalian heart. Total RNA was isolated and PCR with reverse transcription (RT-PCR) products with primer pairs specific to rat *KCNIP*s were visualized by electrophoresis. (**a**) The RT-PCR results of a positive control for *KCNIP1-4* (K1-4) from a representative sample of rat brain. (**b**) The RT-PCR results of representative left atrium (LA), right atrium (RA), left ventricle (LV) and right ventricle (RV). *KCNIP1-3* mRNAs could be detected in the mammalian LA, RA, LV and RV. GP, glyceraldehyde 3-phosphate dehydrogenase; M, molecular weight maker. Data are representative of three independent experiments. Full-length blots are presented in Supplementary Fig. 4.

**Figure 2:**
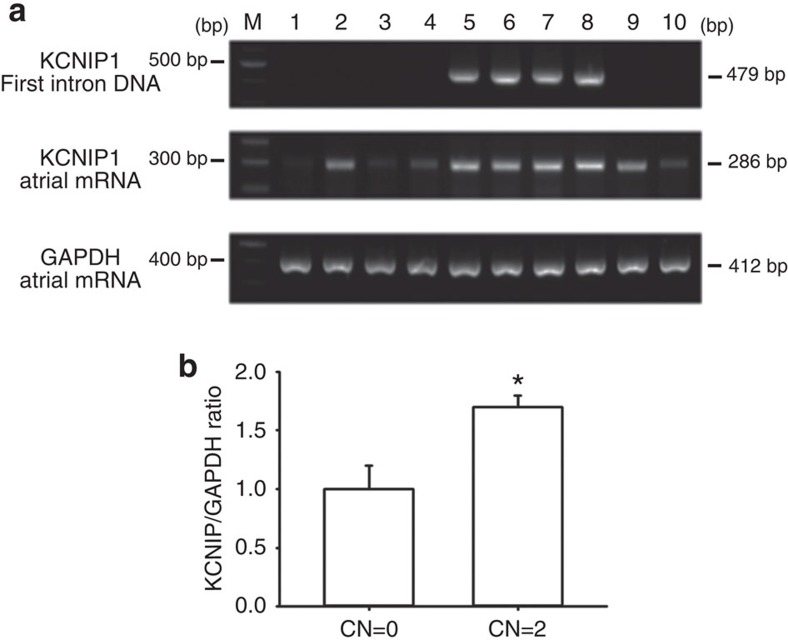
Individuals with insertion in intron 1 of *KCNIP1* have a higher *KCNIP1* mRNA expression. (**a**) Top panel: The detection of insertion/deletion in intron 1 of human *KCNIP1* was performed by PCR amplification of genomic DNA with primers targeting the sequences within the CNV segment in intron 1 of human *KCNIP1*. No PCR band indicates homozygous deletion (copy number (CN)=0) (patients 1–4, 9 and 10); the presence of one PCR band (479 bp) indicates homozygous insertion (CN=2)(patients 5–8). Middle and lower panels: *KCNIP1* and *GAPDH* mRNA expressions were semiquantified by PCR with reverse transcription and visualized by electrophoresis. White blood cell mRNA samples from those with *KCNIP1* intron homozygous insertion (patients 5–8) show higher PCR band density, indicating a higher *KCNIP1* mRNA level. Arrows indicate the locations of PCR bands. (**b**) Quantification of *KCNIP1* expression (normalized to GAPDH) in patients with CN=0 and those with CN=2. Data are representative of three independent experiments. Error bars, s.d. Mann–Whitney *U*-test; *P<0.05. Full-length blots are presented in Supplementary Fig. 5.

We then tried to investigate the expression patterns of the four *KCNIP* mRNAs in the mammalian heart. We found that *KCNIP1-3* are expressed in all the four chambers of the heart (Fig. 1). Interestingly, we observed that the CNV pattern in the first intron of the *KCNIP1* gene significantly affected the expression level of *KCNIP1* mRNA (Fig. 2). Homozygous insertion was associated with a significantly higher *KCNIP1* mRNA level. This result indicates that a higher *KCNIP1* expression or higher KCHIP1 level/function may be associated with the mechanism of AF and increased susceptibility to AF. In the next step, we tried to address this possibility in animal studies.

### Effect of *KCNIP1* knockdown and overexpression in hearts

Because *KCNIP1* is expressed in the atrium, in the next step, we sought to investigate the role of KCHIP1 in the atrium. Recently, zebrafish has become a powerful vertebrate genetic model system used to study the mechanism of cardiac arrhythmia because its fundamental electrophysiological properties, such as heart rates, action potential morphology and electrocardiogram morphology, are remarkably similar to those of human^19^. Therefore, in the present study, instead of mice which have extremely short action potential durations (APDs), we used zebrafish to investigate the functional role of KCHIP1 in the mechanism of AF.

APD shortening at high rates is critical to maintain AF and is the major electrophysiological mechanism of AF^20^. Calcium plays a pivotal role in the mechanism of AF, because calcium overload is common at high rates in AF^21^. KCHIP1 is a calcium-binding protein and may mediate the interplay between intracellular calcium and modulation of potassium currents, which are the major repolarization currents and are essential to determine APD^22^. Therefore, it is logical to speculate that KCHIP1 may play an important role in the mechanism of APD shortening during AF. We then sought to evaluate the response of APD shortening at high rates when the *KCNIP1* was knocked down or overexpressed, using zebrafish as the genetic model system.

There are two homologues of *KCNIP1* (*KCNIP1a* and *KCNIP1b*) in zebrafish. The sequence of *KCNIP1a* is largely unknown, and *KCNIP1b* shares much sequence homology with mammalian *KCNIP1*. In the present study, our zebrafish experiment targeted on *KCNIP1b* (zebrafish chromosome 10). We have screened the CN of the zebrafish *KCNIP1b*, and only one gene copy was noted. Whole-mount *in situ* hybridization shows that the expression of *KCNIP1b* is first detected on the third day post fertilization (Supplementary Fig. 3a). Grossly, *KCNIP1b* MO or mRNA overexpression did not cause significant developmental delay in the zebrafish (Supplementary Fig. 3b).

**Figure 3:**
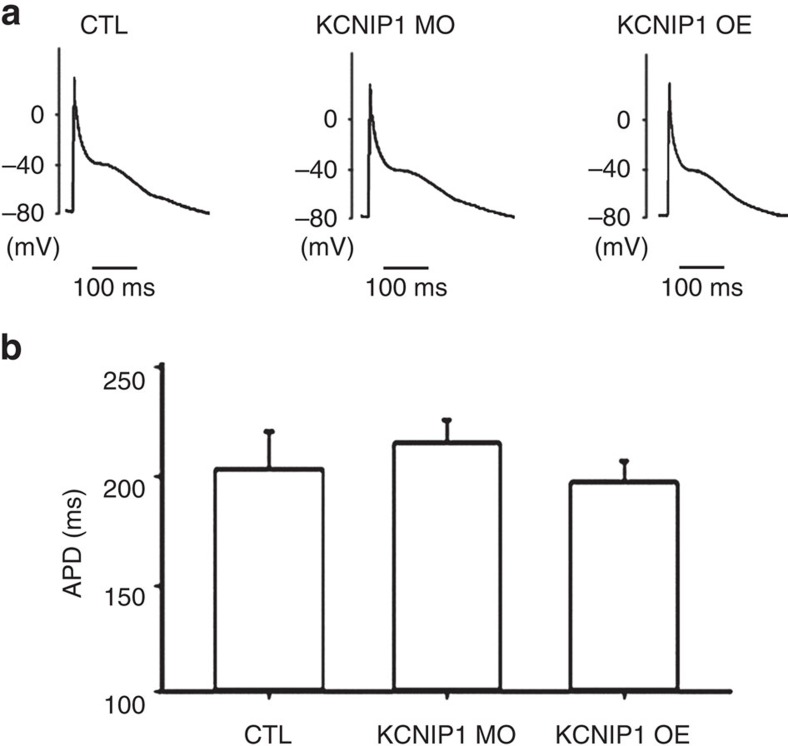
Atrial APDs of KCNIP1-knockdown or overexpression hearts are comparable to control hearts. (**a**) Representative atrial action potentials of *KCNIP1*-knockdown (KCNIP1 MO), overexpression (KCNIP1 OE) and control (CTL) hearts at a baseline pacing rate of 188 beats per min. Action potentials were recorded by using the microelectrode and disrupted patch method. (**b**) Quantification of atrial APD at 90% repolarization. The mean APD was comparable between KCNIP1 MO, KCNIP1 OE and the CTL hearts. *N*=3 experiments for each group. Error bars, s.d. Mann–Whitney *U*-test.

Regarding cardiac and electrophysiological phenotypes, the heart rates were comparable between control, *KCNIP1* knockdown and overexpression hearts (178±15 versus 171±20 versus 175±19 beats per min for control, knockdown and overexpression hearts, respectively). The baseline atrial action potentials for the three groups are shown in Fig. 3a. There was no significant change of the atrial APD (204±14 versus 213±9 versus 192±10 ms for control, knockdown and overexpression hearts, respectively) (Fig. 3b).

**Figure 4:**
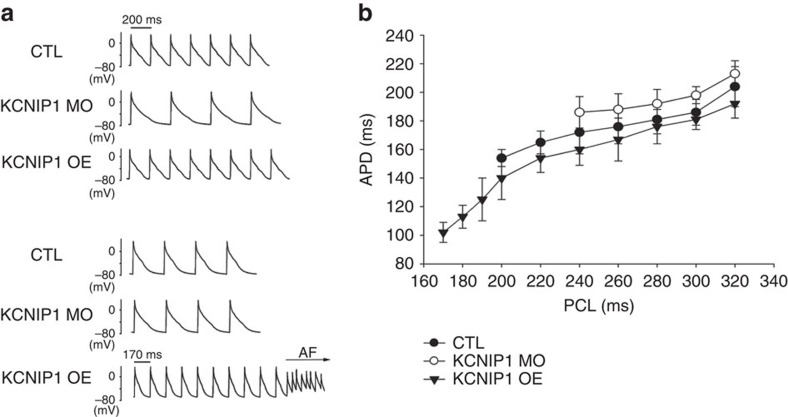
*KCNIP1*-knockdown and overexpression modulates APD shortening at high atrial rates. (**a**) Representative atrial action potential tracings at increasing pacing rates for *KCNIP1*-knockdown (KCNIP1 MO), overexpression (KCNIP1 OE) and control (CTL) hearts are shown. At pacing cycle length (PCL) 200 ms, the CTL and *KCNIP1*-overexpression hearts could sustain 1 to 1 pacing rate, but the *KCNIP1*-knockdown heart could not, and 2 to 1 capture is noted, indicating that the *KCNIP1*-knockdown heart could not maintain as higher rates as the CTL heart. At even shorter PCL (170 ms), only the *KCNIP1*-overexpression heart could sustain 1 to 1 high pacing rate, but the *KCNIP1*-knockdown and CTL hearts could not (2 to 1 capture), indicating that the *KCNIP1*-overexpression heart could maintain higher rates than the CTL heart. Interestingly, at this high-rate pacing, atrial tachyarrhythmia or AF could be induced in the *KCNIP1*-overexpression heart. (**b**) Summary data for three independent experiments demonstrating the relationship of PCL and APD from CTL, *KCNIP1*-knockdown and overexpression hearts are shown. With increasing rate (decreased PCL), APD shortens accordingly to facilitate maintenance of high rate in all the three groups. However, less APD shortening is observed in the *KCNIP1*-knockdown hearts. The maximal pacing rate is lower in the *KCNIP1*-knockdown hearts and higher in the *KCNIP1*-overexpression hearts, compared to that of the CTL hearts. *N*=3 experiments for each group. Error bars, s.d.

However, hearts with *KCNIP1* knockdown showed less atrial APD shortening at increasing rates and could not sustain high atrial pacing rates as compared with control hearts. On the contrary, hearts with *KCNIP1* overexpression showed more atrial APD shortening at increasing rates and could sustain higher atrial pacing rates as compared with control hearts (Fig. 4). Interestingly, one transient atrial tachycardia or AF could be induced in the *KCNIP1* overexpression hearts during high-rate pacing (Fig. 4). AF could not be induced in control and *KCNIP1* knockdown hearts.

### Interaction of KCHIP1 protein with cardiac ionic channels

Previously KCHIP2 had been identified as the cardiac KCHIP assembled in macromolecular structures with the Kv4.2 channel to generate and regulate the cardiac transient outward current^16^,^17^. Recently it has also been shown that KCHIP2 is also associated with the Cav1.2 channel and modulate the cardiac L-type calcium current^23^. Whether *KCNIP1*-encoded protein KCHIP1 also interacts with cardiac ionic channels, such as Kv4.2, Kv4.3 or Cav1.2 in the atrium has never been investigated before.

**Figure 5:**
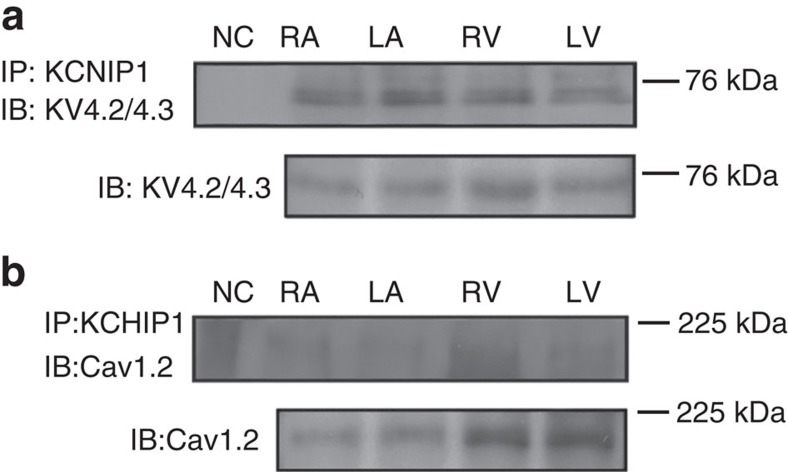
Co-immunoprecipitation shows a biochemical association between KCHIP1 and KV4.2/4.3. (**a**,**b**) Tissue samples from adult rat heart were lysed and immunoprecipitated (IP) with anti-KCHIP1. The membranes were then immunoblotted (IB) using anti-KV4.2/4.3 (**a**) and anti-Cav1.2 (**b**). Beads conjugated with IgG isotype were used as negative control (NC). There is a biochemical association between KCHIP1 and KV4.2/4.3 (**a**), but no association between KCHIP1 and Cav1.2 (**b**). Data are representative of three independent experiments. Full-length blots are presented in Supplementary Fig. 6.

Using co-immunoprecipitation, we demonstrated that KCHIP1 was also physically associated with the Kv4.2/4.3 proteins (Fig. 5a). However, not like KCHIP2, KCHIP1was not physically associated with the Cav1.2 protein in the atrium (Fig. 5b). These results raise the possibility that KCHIP1 can also modulate atrial transient outward current and thus modulate atrial repolarization properties, as shown in the zebrafish experiments. In the next step, we tried to address this issue using cultured atrial myocytes.

### Effect of KCHIP1 in atrial myocytes

**Figure 6:**
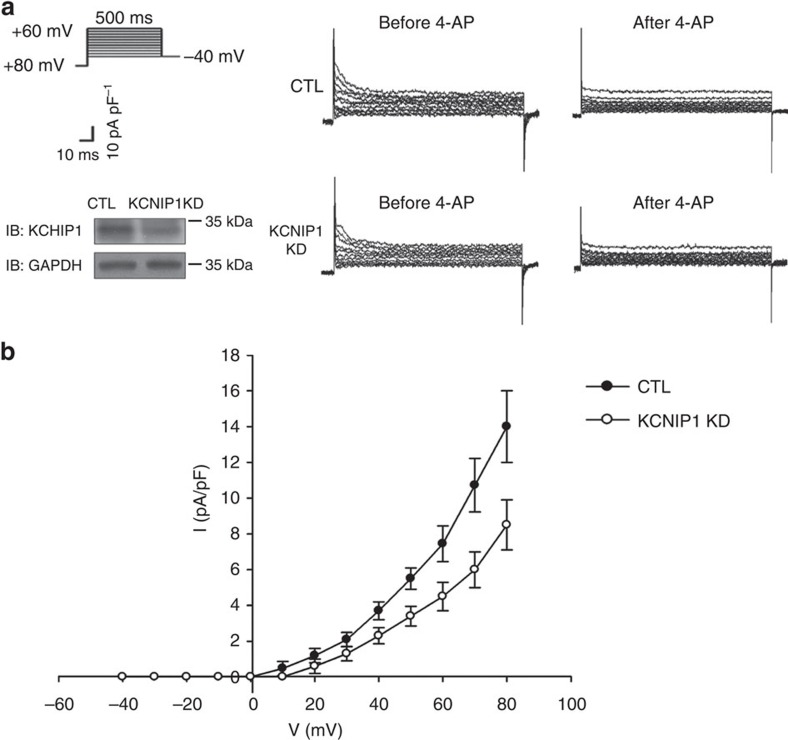
Knockdown of *KCNIP1* downregulates transient outward currents in atrial myocytes. (**a**) Transient outward current (Ito) was obtained by a family of depolarization steps from −80 mV, and was measured as the 4-aminopyridine (4-AP)-sensitive peak current (10 mM). (left upper) Voltage protocol. Right panel shows the representative recordings of 4-AP sensitive currents of the control (CTL) and *KCNIP1* knockdown (KCNIP1 KD) atrial myocytes, respectively. (left lower) Efficiency of knockdown represented by decreased KCHIP1 protein level. (**b**) Representative current density–voltage relationships of 4-AP sensitive currents in CTL and *KCNIP1* KD atrial myocytes. *N*=3 experiments for each group. Error bars, s.d. Full-length blots are presented in Supplementary Fig. 7.

Kv4.2 and Kv4.3 are the major pore-forming subunits of the cardiac transient outward current^13^. We sought to investigate the effect of KCHIP1 knockdown or overexpression on the cardiac transient outward current. To address this issue, we used a murine atrial cell line HL-1, which is the only available atrial myocyte cell line that continuously divides, maintains a differentiated atrial phenotype with spontaneous depolarization and has a high transfection efficiency for genetic manipulation^18^,^19^,^20^. We found that knockdown of *KCNIP1* significantly impaired the cardiac transient outward current (Fig. 6a,b). We failed to overexpress *KCNIP1* because of the low efficiency of the expression plasmid (no significant change of KCHIP1 protein level after overexpression). Nevertheless, this result indicates that the *KCNIP1*-encoded protein KCHIP1 also modulates atrial potassium currents and, thus, may play a role in modulating atrial repolarization.

## Discussion

This is the first study to investigate the role of genomic CNV in determining the susceptibility to AF and is also the first genome-wide CNV study of AF. We identified a common CNV in human *KCNIP1* gene that was a strong genetic predictor of AF. We demonstrated that intronic CNV in the human *KCNIP1* gene determined the mRNA level of *KCNIP1*, and *KCNIP1*-encoded protein KCHIP1 was linked to the mechanism of maintaining higher atrial rates, and is the possible future target for AF treatment.

*KCNIP1*-encoded protein KCHIP1 is a KCHIP and also a calcium-binding protein. It was traditionally thought an auxiliary subunit of the protein complex Kv4.3 potassium channel in neurons, and modulates the neural voltage-gated A-type Kv4.3 current^18^. In the present study, we first demonstrated the expression of *KCNIP1* in the mammalian atrium. Previously, it has been demonstrated that KCHIP2 is the main cardiac KCHIP (mainly in ventricles), and KCHIP2 is a subunit protein of Kv4.2 and Cav1.2, modulating the ventricular transient outward potassium current and L-type calcium current, respectively^23^. In the present study, we first demonstrated that in the mammalian atrium, KCHIP1 formed a protein complex with Kv4.2/4.3 and modulated the atrial transient outward current, thus modulating atrial repolarization properties.

KCHIP1 is a calcium-binding protein. The intracellular calcium level plays a pivotal role in the mechanism of AF^24^,^25^. At high atrial rates, the intracellular calcium level is accumulated and elevated due to the short time available for beat-to-beat diastolic calcium reuptake into the sarcoplasmic reticulum, thereby triggering atrial electrical remodelling (for example, APD shortening) and facilitating the maintenance of AF. Our results first provide the possibility that KCHIP1 mediates the regulation of potassium channel by calcium in the mammalian atrium at high rates. It may be speculated that in response to elevated intracellular calcium at high atrial rates, KCHIP1 augments transient outward potassium current and shortens APD. Accordingly, in the zebrafish model, we have shown that genetic overexpression of *KCNIP1* resulted in facilitation of APD to shorten at high rates and promotes AF. The results of our human genetic study are also compatible with this finding because most of the AF patients harboured a *KCNIP1* intron insertion, which is associated with higher *KCNIP1* expression.

The results of the present study also suggest that targeting on KCHIP 1 may be a future therapeutic approach to treat human AF. As mentioned before, we have demonstrated that overexpression of *KCNIP1* may predispose to AF, implicating that inhibiting *KCNIP1*-encoded protein KCHIP1 may prevent AF. Because currently there has been no specific inhibitor of KCHIP1, developing anti-*KCNIP1* RNA interference may be an option, and testing of this gene therapy in human clinical trials are warranted in the future to demonstrate the efficacy of inhibiting *KCNIP1* expression to treat AF.

Four *KCNIP* genes have been identified in humans and mice, encoding KCHIP1-4 (refs 16, 17). There are also four *KCNIP* genes identified in zebrafish (sources from ZFIN). A well-known phenomenon in the cardiac electrophysiology is the compensatory mechanism to prevent perturbation of the normal electrophysiological function, even when a function of an ionic channel or its regulatory protein is markedly altered^26^. Therefore, since the four KCHIP paralogous proteins may function similarly to modulate the transient outward potassium current in the heart, it is logical to speculate that overexpression of *KCNIP1* may trigger compensatory downregulation of the functions of other paralogous proteins, resulting in minimal change of the transient outward potassium current. Unfortunately, it is difficult to isolate single atrial cardiomyocytes from the small zebrafish embryo atrium with *KCNIP1* overexpression and measure the transient outward potassium current. Furthermore, we did not have the data of the expression of other paralogues. We could only evaluate gross action potential morphology or measure APD of the intact whole heart, and did find no significant change of resting action potential morphology or APD in the heart with *KCNIP1* overexpression. However, we found that the change of APD could manifest at extreme conditions which the compensatory mechanism could not keep pace with, such as at very high rate. Therefore, we could only find phenotypic change of APD by high-rate pacing.

There are several studies using a zebrafish model for the genetic studies of AF^27^,^28^,^29^,^30^. Müller *et al*.^27^ knocked down a candidate gene, *GREM2*, in zebrafish, and the morphants showed abnormal atrial development/differentiation. Overexpression of mutant *GREM2* resulted in abnormal atrial rhythm^27^. Liang *et al*.^28^ knocked down candidate genes *kcnk3a* and *kcnk3b*, which resulted in lower heart rate and marked increase in atrial diameter. Sinner *et al*.^29^ knocked down genome widely identified *NEURL* and *CAND2* genes, and the morphants showed prolonged atrial APD. All these phenotypes are surrogate phenotypes of AF or substrates that may predispose to the development of AF, but not real AF phenotype. The only studies that showed real electrophysiological AF were Rottbauer's study^30^ and our study. Rottbauer *et al*.^30^ first reported a zebrafish model with loss of L-type calcium channel function (isl mutant), and spontaneous AF was recorded in ECG. In our study, we induced AF in one *KCNIP1* (a GWAS-identified gene) overexpressed zebrafish.

There are several limitations in our study. First, although most of the neighbouring SNPs were not better than the CNV in predicting AF risk and CNV genotypes predicted *KCNIP1* expression level (probably functional or disease causing), we still could not rule out the possibility that there is another true disease causing variant. We did not find the exact breaking points of the CNV. An intense effort to map the exact CNV breakpoints and dense fine mapping of SNPs around the breakpoints might well identify better SNP markers or even the disease causing variant. Second, we did not address all the molecular mechanisms experimentally. To address the full molecular mechanisms, how the CNV affects the expression of *KCNIP1*, the physiological function of *KCNIP1*-encoded protein KCHIP1 in the atrium and how loss of function or gain of function predisposes to AF in an animal model should be addressed. Although we showed that the CNV affects the expression level of *KCNIP1* mRNA, we did not show the mechanism by which this CNV regulates *KCNIP1* expression. The lengths and sequences of intron 1 of the *KCNIP1* gene are markedly different between fish and humans, and this 4-kb CNV segment found in humans does not exist in zebrafish. To study the specific function of this 4-kb CNV segment, state-of-the art technique CRISPR or TALEN may be adopted in large mammals that may also have this specific intron segment. We have experimentally shown that *KCNIP1*-encoded protein KCHIP1 is physically associated to atrial Kv proteins and modulates atrial transient outward current, which plays an important role in atrial electrophysiology. We have also experimentally proved that KCHIP1 mediates facilitation of APD shortening at high rates which may predispose to AF. Therefore, we only addressed part of the molecular mechanism experimentally. Finally, we could not rule out the possibility that *KCNIP1* (also expressed in neurons) is linked to AF through a neurogenic mechanism.

In conclusions, the present study is a translational research in which we directly translated the results of genetic and molecular studies into a new idea of AF disease mechanism, which further implicates a therapeutic potential of targeting on KCHIP1 to treat human AF in the future.

## Methods

### Study population

The study participants were from the National Taiwan University AF Registry (Northern Taiwan). We used a three-stage study design with the attempt to minimize false positive findings yet maximize power and efficiency by examining samples with gradually increased phenotypic severity but with increasing sample size^9^. In stage I, we selected 50 younger AF patients with extremely severe phenotype, who were diagnosed as having symptomatic persistent AF with very frequent AF attacks (≥ one attack per day), and no identifiable underlying cardiovascular diseases responsible for the cause of AF (lone AF). Genotyping these patients with disease phenotypes at the extremes is more likely to identify underlying genetic causes although the case number may be small due to the lower prevalence of these patients^9^. The healthy controls had NSR and had no identifiable cardiovascular diseases.

In stage II, we selected another 105 symptomatic persistent lone AF patients with less attack frequency than the stage I patients (≥ one AF attack per week), and 422 NSR healthy controls to replicate the significant CNVs in stage I. In stage III, we replicated the significant CNVs in both stages I and II in a combined larger population. The significant CNV was also validated in 275 AF cases and 546 NSR controls from different geographic areas (Middle and Southern Taiwan) in stage III.

The criteria for selection of case and control patients have been reported previously^31^,^32^,^33^,^34^. Cases were patients with AF, whereas controls were those with NSR. Patients with AF due to hyperthyroidism were excluded. We also included those with highly suspicious but non-documented AF and those with other atrial arrhythmia for the large stage III population. Informed consent was obtained from participating subjects and the study was approved by the institutional review board of the National Taiwan University Hospital. The clinical data for the whole study participants are provided in Supplementary Table 1. The clinical data for the study participants in the Middle and Southern Taiwan are provided in Supplementary Table 2.

### Genome-wide detection of CNV

Using the Illumina HumanOmni1-Quad BeadChip (1,140,419 markers) SNP-based probes and additional CNV intensity probes, genome-wide genotyping was performed to obtain signal and allelic intensities and CNV regions were first identified through comparing with HapMap controls^35^,^36^. To increase the sensitivity of CNV identification, we incorporated multiple factors using PARTEK Genomic Suite 6.6 (PARTEK. Inc., St Louis, USA), a program that is based on the segmentation algorithm. The criteria for the detected CNV segments were as previously reported criteria^35^,^36^: (1) neighbouring regions with significantly different average intensities, and the significant level of *P* value <0.001; (2) breakpoints (region boundaries) that yielded the optimal statistical significance (smallest *P* value); and (3) signal-to-noise ratio ≥0.3. SNPs with a smoothing value below and above 2±0.4 were considered loss and gain, respectively. The approximate CNV breakpoints were predicted *in silico*, and because the density of the SNPs in the Chip is sparse, the breakpoint is defined at the midpoint between two distinct genomic segments. These two genomic segments are also *in silico* predicted. When the average allelic intensity of two regions are significant different, they are defined as two distinct regions or two distinct segments and a CNV region is called. Then the breakpoint is defined at the midpoint between the two adjacent boundaries of the two distinct segments. The size of the CNV is defined as the genomic length between the 5′ and 3′ breakpoints. We identified a total of 7,210 CNVs. NCBI RefSeq (hg18; build 36) was used to annotate the location and coding region of each CNV region in the genome.

### Verification of CNV regions

Quantitative PCR (qPCR) and semiquantitative conventional PCR with densitometry were performed to verify the CNV and determine the CN of the targeted gene (for example, *KCNIP1*) in the study samples. qPCR was performed with the TaqMan Copy Number Reference Assay RNase P following the TaqMan qPCR protocol. Each 15-μl reaction mixture comprised 50-ng genomic DNA and 1 TaqMan probe/primer mix in 1 TaqMan Master Mix, amplified on an Applied Biosystems QuanStudio 3D. qPCR data were collected and analysed by the ABI software (Life Technologies Co., CA, USA). Semiquantitative conventional PCR with densitometry were performed for large samples with designed primers targeting the sequences within the postulated deletion segment. There was no PCR product from those samples with zero copy and presence of PCR band from those with more or equal to one copy.

### Zebrafish experiments

Breeding and maintenance of TL strain zebrafish, as well as collecting and staging of embryos, were done according to standard procedures^37^. Some embryos were reared in egg water treated with 0.003% 1-phenyl-2-thiourea to inhibit pigmentation^37^. Developmental times refer to days post-fertilization. All embryos were observed and photographed at specific stages under a microscope (MZFLIII, Leica) equipped with Nomarski differential interference contrast optics and a CMOS digital camera (Canon EOS-1DX)^37^. Zebrafish embryos were obtained by natural mating. MO or *in vitro* synthesized mRNA microinjection was performed at the stage of 1–4 cells. The *KCNIP1b*-MO antisense oligonucleotide was designed to direct against the 5′ untranslated region (transcription start site) of the *KCNIP1b* gene (5′-TCAATGTGCCCACTACTGCTCCCAT -3′). Capped zebrafish *KCNIP1* mRNA was synthesized with the mMESSAGE mMACHINE kit (Ambion Inc., Austin, TX). Embryos positioned in an agarose injection chamber were injected using a Narishige micromanipulator and needle holder (Narishige, Tokyo, Japan). The zebrafish experiments were approved by the Institutional Animal Care and Use Committee of the National Taiwan University College of Medicine.

### Whole-mount *in situ* hybridization

Whole-mount *in situ* hybridization was performed as our standard protocols^37^. The *KCNIP1b* probe was prepared from transOMIC clone BC086698 using primers PME18S F (5′-TGTACGGAAGTGTTACTTCTGCTC-3′) and PME 18S rev T3(5′-GGATCCATTAACCCTCACTAAAGGGAAGGCCGCGACCTGCAGCTC-3′) to prepare the template and T3 RNA polymerase for RNA synthesis. Embryos were fixed overnight at 4 °C in 4% paraformaldehyde buffered with 1 × phosphate-buffered saline. After permeabilization, embryos were hybridized overnight. Then embryos were incubated with anti-DIG antibody conjugated to AP, and developed with NBT-BCIP reagents.

### Zebrafish embryo heart electrophysiological recordings

The heart of zebrafish embryos 3 days after fertilization was dissected from the thorax *en bloc* by using fine forceps and transferred to the recording chamber. Only spontaneously beating whole hearts were studied. All experiments were performed at room temperature. The recording chamber was superfused with a solution containing 140 mM NaCl, 4 mM KCl, 1.8 mM CaCl_2_, 1 mM MgCl_2_, 10 mM glucose and 10 mM Hepes (pH 7.4)^19^,^37^. Action potentials were recorded by using the microelectrode and disrupted patch method^19^,^37^. Atrial action potentials were measured by using an amplifier (Axopatch 200B; Axon Instrument, USA) and digitized with a 12-bit analogue-to-digital converter (Digidata 1440A Interface; Molecular Devices, USA). Resting action potentials were first validated and then triggered by incrementally injecting pulses of depolarizing current or field pacing. APD was measured at 90% repolarization^37^,^38^.

### Reverse transcription polymerase chain reaction

The extraction and quantification of mRNA by means of reverse transcription polymerase chain reaction (RT-PCR) were performed as our standard protocols^24^,^25^,^38^,^39^,^40^. Total RNA was extracted from the cardiac tissues of male Wistar rats (weight 300±20 g, aged 3–4 months). The experimental protocol conformed to ref. 41 and was approved by the Institutional Animal Care and Use Committee of the National Taiwan University College of Medicine. The tissue was homogenized with a Polytrone-Aggregate (Dispergierund Mischtechnik, Littau, Switzerland), and Trizol solution (Gibco BRL, Grand Island, NY) was added for RNA extraction. The extracted RNA was dissolved in diethyl pyrocarbonate-treated distilled water. Spectrophotometry at 260 and 280 nm was performed to measure the amount and quality of RNA. The RNA was then converted to complementary DNA by reverse transcription with random hexanucleotides and avian myeloblastosis virus reverse transcriptase (Boehringer, Mannheim, Germany). The primers are as follows: *KCNIP1*: forward: 5′-CGACCCTCCAAAGATAAGATTG-3′, reverse: 5′-AGTTCCTCTCAGCAAAATCGAC-3′; *KCNIP2*: forward: 5′-GACTTTGTGGCTGGTTTGTC-3′, reverse: 5′-ATGGTCACCACACCATCCTT -3′; *KCNIP3*: forward: 5′-ATTTACGCGCAGTTCTTCCC-3′, reverse: 5′-GTAGCCATCCTTGTTAATGTC-3′; *KCNIP4*: forward: 5′-AGCGTGGAAGATGAACTGGA-3′, reverse: 5′-CCTGTGGAAAGAACTGCGAG-3′.

Single-stranded complementary DNA was amplified with PCR. The PCR products were confirmed by means of direct sequencing. The *glyceraldehyde 3-phosphate dehydrogenase (GAPDH)* gene was used as the internal control for equal loading. The reaction products were analysed with agarose gel electrophoresis. Optic densitometry was performed after the gel was stained with ethidium bromide for semiquantitative measurements of the DNA amount^24^,^25^,^38^,^39^,^40^. The SYBR green method was used for quantitative measurement if necessary^24^,^25^,^38^,^39^,^40^. Expression of mRNA was represented by its ratio to the mRNA of GAPDH.

### Immunoprecipitation and immunoblotting assays

Preparations for protein extracts and western blot analyses were performed according to our standard protocols^24^,^25^,^38^,^39^,^40^. Total proteins were extracted from the cardiac tissues of male Wistar rats (weight 300±20 g, aged 3–4 months) according to the manufacturer's instructions (Chemicon Compartment Protein Extraction Kit, Millipore, MA, USA). The experimental protocol conformed to the *Guide for the Care and Use of Laboratory Animals* (NIH Publication, 8th edition, 2011) and was approved by the Institutional Animal Care and Use Committee of the National Taiwan University College of Medicine. The primary antibodies used in the present study included rabbit polyclonal anti-KCHIP1 (1:500; Alomone Labs), rabbit polyclonal anti-Cav1.2 (1:5,000; Alomone Labs) and rabbit polyclonal anti-Kv4.2/4.3 (1:500; Santa Cruz). Rabbit peroxidase-conjugated secondary antibodies (1:4,000; Santa Cruz) were used for detection of the primary antibody.

For immunoprecipitation^39^, protein lysates were incubated with 1 μg ml^−1^ of the anti-KCHIP1 antibody (Alomone Labs) overnight at 4 °C. Immunocomplexes were collected by incubation with 50 μl of protein A for 2 h. Immunoprecipitates were washed four times with ice-cold lysis buffer and the pellets were re-suspended in 2 × sample buffer. The samples were then subjected to SDS–polyacrylamide gel electrophoresis and immunoblotted with an anti-Kv4.2/4.3 (1:500; Santa Cruz) or anti-Cav1.2 (1:5,000; Alomone Labs) antibodies. The proteins were visualized by enhanced chemiluminescence (Amersham).

### HL-1 atrial myocytes experiments

We used a murine atrial cell line HL-1 to study the effect of genetic manipulation of *KCNIP1* on the change of atrial transient outward current. HL-1 atrial myocyte cell line is the only available atrial myocyte cell line that continuously divides, maintains a differentiated atrial phenotype with spontaneous depolarization and has a high transfection efficiency for genetic manipulation^24^,^25^,^38^,^39^. HL-1 atrial myocytes were cultured as per our standard protocols^24^,^25^,^38^,^39^. HL-1 atrial myocytes were cultured in Claycomb medium supplemented with 10% fetal bovine serum, 100 units per ml penicillin, 100 μg ml^−1^ streptomycin, 0.1 mM norepinephrine and 2 mM L-glutamine. Transient transfection of HL-1 atrial myocytes was carried out using LipofectAMINE 2,000 (Invitrogen) according to the manufacturer's instructions. Transfection efficiency based on the GFP fluorescence was 60–80% for HL-1 cells^24^,^25^,^38^,^39^. Transmembrane currents in HL-1 atrial myocytes were measured by using the whole-cell recording technique with a patch-clamp amplifier (Axopatch 200B; Axon Instrument, USA) and digitized with a 12-bit analogue-to-digital converter (Digidata 1440A Interface; Molecular Devices, USA) as previously described^24^,^25^. Transient outward current (Ito) was obtained by a family of depolarization steps from −80 mV, and was measured as the 4-aminopyridine (4-AP)-sensitive peak current (10 mM).

### Statistical analysis

CNV association analyses were performed using the logistic regression model to adjust non-genetic covariates. The statistical significance level was set at *P*<10^−3^ in the genome-wide discovery stage. We used a more liberal threshold of *P*<10^−3^ in our stage I genome-wide discovery stage because of the small stage I sample size, and relied on subsequent validation in stages II and III with larger samples. Similar strategy with a liberal threshold in the stage I exploratory sample and then followed by critical validation in larger samples has been adopted in several genome-wide studies^9^. Furthermore, recently Panagiotou and Ioannidis^42^ also showed that a substantial proportion of the associations with borderline genome-wide significance represent replicable and possible associations, and suggest a relaxation in the current GWS threshold. Finally, the resulting significant CNVs were excluded if they resided on telomere- or centromere-proximal cytobands or on genomic regions with extremes of GC content, which produces hybridization bias.

The regional plot for the association of SNPs selected from the genome-wide humanOmni1-Quad BeadChip in the region flanking the *KCNIP1* intron 1 CNV, together with the *KCNIP1* intron 1 CNV itself, with AF was created to find possible significant SNPs other than CNV itself. For each SNP, the chromosomal location was shown on the *x* axis and the significance level for association with AF was indicated by a –log_10_P value on the *y* axis. *P* values were expressed as –log_10_(P)(*y* axis) for every tested SNP ordered by chromosomal location (*x* axis). Genomic position was determined using the NCBI database (NSCI Build 36). Data were analysed with the R version 3.1.2 software (The R Project for Statistical Computing).

The statistical significance level was set at *P*<0.05 after Bonferroni correction in the replication stages. Power estimation revealed that we had >95% power to replicate the association for an odds ratio of 2.0 at an alpha level of 0.05 with at least 250 cases and 500 controls in the replication population. Homogeneity of association across stages was tested using the Mantel–Haenszel method and the statistical significance level was set at *P*<0.05.

For the molecular and electrophysiological studies, all data were expressed as mean±s.d. Data from independent group were compared using the Mann–Whitney *U* test for continuous data and Fisher's exact test for categorical data. The statistical significance level was set at *P*<0.05 after Bonferroni correction.

## Additional information

**How to cite this article:** Tsai, C.-T. *et al*. Genome-wide screening identifies a *KCNIP1* copy number variant as a genetic predictor for atrial fibrillation. *Nat. Commun.* 7:10190 doi: 10.1038/ncomms10190 (2016).

## Supplementary Material

Supplementary Information Supplementary Figures 1-7 and Supplementary Tables 1-2

## Figures and Tables

**Table 1:** Significant CNV regions in the stage I discovery sample.

Cytoband	Start position (bp)	Type	Allele frequency	Avg. CN	Length (bps)	Gene	DGV	*P*-value
1q12	141,622,815	Loss	0.21	1.58390	395,766	*ANKRD20A12P*	+	3.09E−07
1p36.13	17,546,966	Loss	0.24	1.52439	4,858	*PADI4*	+	2.94E−06
1p36.21	13,306,366	Loss	0.23	1.57085	192,072	*PRAMEF14*	−	1.73E−06
1p36.21	13,167,437	Loss	0.22	1.56805	130,829	*PRAMEF3*	+	1.12E−07
1p36.32	2,573,413	Loss	0.22	1.55573	108,341	*TTC34*	+	1.03E−05
2p16.1	56,378,184	Loss	0.23	1.54534	120,493	*CCDC85A*	+	5.83E−07
2p22.3	33,077,316	Loss	0.23	1.54556	3,928	*LTBP1*	+	1.02E−03
2p25.3	1,506,306	Loss	0.23	1.59170	16,162	*TPO*	+	2.61E−07
3q29	199,289,792	Loss	0.25	1.51397	142,634	*ANKRD18DP*	+	6.27E−08
5q35.1	170,061,229	Gain	0.29	2.57862	4,470	*KCNIP1*	+	1.57E−06
6p21.32	33,140,517	Loss	0.22	1.56019	8,849	*HLA-DPA1*	+	1.88E−06
6p21.32	33,133,423	Loss	0.22	1.56438	7,095	*HLA-DPA1*	+	1.00E−06
6p21.32	33,158,459	Loss	0.21	1.57858	4,840	*HLA-DPB1*	+	7.36E−07
6p22.3	22,156,930	Loss	0.30	1.43742	4,981	*LINC00340*	−	3.94E−05
7q34	142,107,052	Loss	0.21	1.57438	69,930	*PRSS1*	+	1.58E−06
7q34	142,107,052	Loss	0.21	1.57438	69,930	*PRSS3P2*	+	1.58E−06
8q24.3	144,785,898	Loss	0.24	1.52527	27,257	*ZNF623*	+	3.63E−05
14q32.33	105,491,658	Gain	0.55	3.10113	19,535	*ADAM6*	+	5.23E−07
14q32.33	105,807,945	Gain	0.33	2.51647	37,608	*LINC00226*	+	3.38E−08
17p13.3	670,214	Loss	0.26	1.47994	2,402	*NXN*	+	1.79E−08
21q22.3	46,142,957	Loss	0.30	1.39238	27,264	*PCBP3*	+	1.56E−07
21q22.3	43,780,913	Loss	0.23	1.53278	23,647	*HSF2BP*	+	2.12E−04
21q22.3	46,171,463	Loss	0.31	1.37456	29,943	*PCBP3*	+	2.48E−07
21q22.3	43,728,391	Loss	0.23	1.54411	52,523	*HSF2BP*	+	7.05E−04
22q11.22	21,430,797	Gain	0.30	2.44319	139,593	*MIR650*	+	9.66E−08
22q11.22	21,430,797	Gain	0.30	2.44319	139,593	*IGLL5*	+	9.66E−08
22q11.23	22,694,904	Loss	0.39	1.22954	33,941	*GSTT1*	+	3.90E−08
22q11.23	22,629,805	Loss	0.37	1.25476	40,795	*GSTT2*	+	6.34E−07

Avg. CN, average copy number; bp, base pair; CNV, copy number variation; DGV, Database of Genomic Variants (http://projects.tcag.ca/variation/). Allele frequencies are inferred from the averaged CN based on a diallelic assumption for each variant.

The statistical significance level was set at *P*<10^−3^ in the genome-wide CNV discovery stage; NCBI RefSeq (hg18; build 36) was used to annotate the location and coding region of each CNV region in the genome.

**Table 2:** Genotype distributions of the human *KCNIP1* gene insertion/deletion polymorphism in the study populations.

Genotype	AF (*N*=105)	NSR (*N*=422)	*P*-value*
*Genotype distribution in the stage II population*			
*D/D*†	41	261	0.289/0.527
*D/I, I/I*†	64	161	2.47 × 10^−6^

Genotype	AF (*N*=1,022)	NSR (*N*=2,025)	*P*-value*
*Genotype distribution in the stage III population*			
*D/D*	426	1,217	0.514/0.867
*D/I, I/I*	596	808	5.38 × 10^−23^

Genotype	AF (*N*=275)	NSR (*N*=546)	*P*-value*
*Genotype distribution in subjects from different geographic areas*			
*D/D*	104	322	0.357/0.234
*D/I, I/I*	171	224	1.23 × 10^−9^

AF, atrial fibrillation; D, deletion allele (∼4,470 bp) in the first intron of the human *KCNIP1* gene; I, insertion allele; NSR, normal sinus rhythm; SNP, single-nucleotide polymorphism.

The insertion (or gain) and deletion denote presence (insertion) and absence (deletion) of the same ∼4 kb chromosomal segment in the intron 1 of the *KCNIP1* gene, respectively. This is a diallelic variant (same as an SNP). In the Taiwanese population, the deletion allele frequency is more than 0.5, and therefore the variant allele or minor allele is the insertion allele (gain in Table 1).

^*^P values for Hardy–Weinberg equilibrium in AF (left-sided value in the top row) and NSR (right-sided value in the top row) populations, and comparison of genotype distribution between AF and NSR populations (bottom row).

^†^*D/D*, homozygous deletion or copy number=0; *D/I*, heterozygous deletion or insertion or copy number=1; *I/I*, homozygous insertion or copy number=2.
