# Supplementary material for: Genome-wide screening identifies a KCNIP1 copy number variant as a genetic predictor for atrial fibrillation
Source: Nat Commun. 2016 Feb 2;7:10190. doi: 10.1038/ncomms10190 (PMC4740744; doi:10.1038/ncomms10190)
Supplement: Supplementary Information — Supplementary Figures 1-7 and Supplementary Tables 1-2 [file ncomms10190-s1.pdf]

Supplementary Information

Supplementary Figure 1

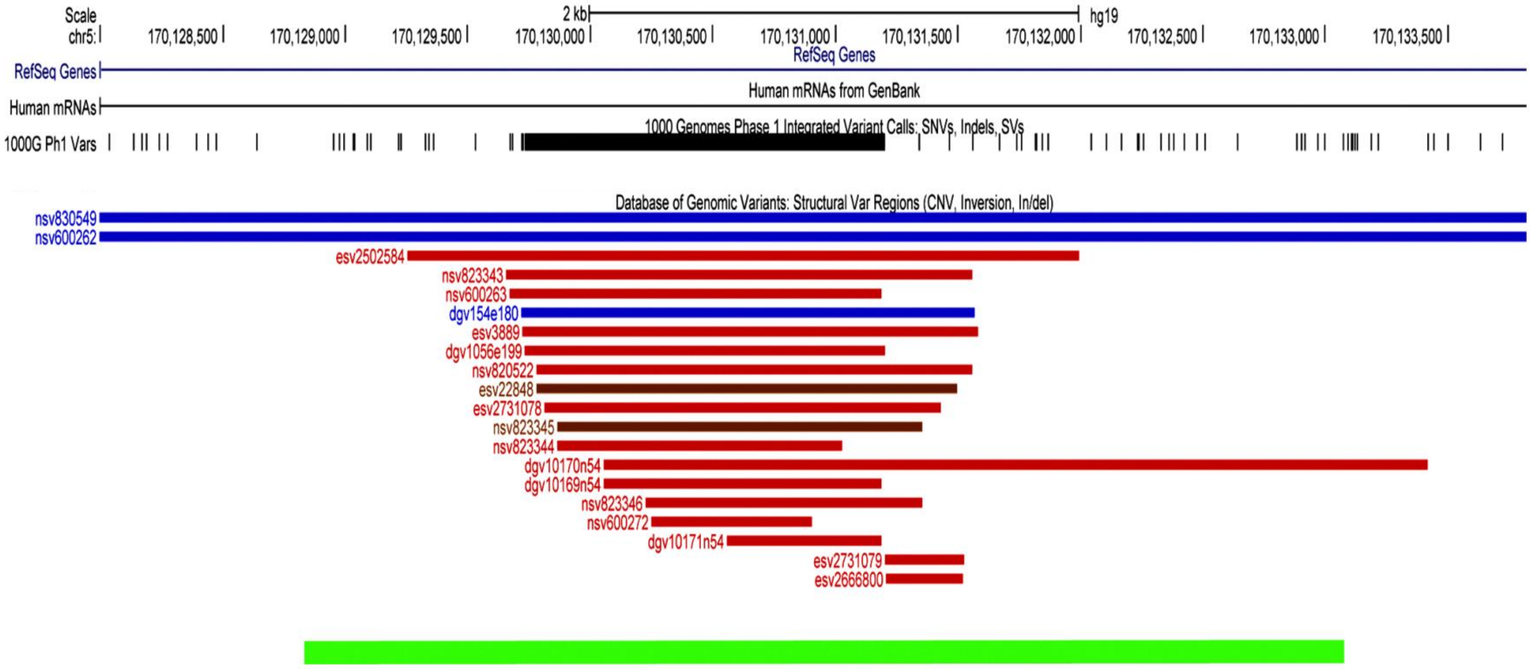

Supplementary Figure 1. The discovered CNV region overlaps with previously reported CNV regions in the Database of Genomic Variants (DGV) and 1000 Genomes Phase1 Integrated Variant Calls. In DGV, Deletions are represented in red, insertions in blue and mixed complex pattern in brown. The corresponding indels variant call in 1000 Genomes is represented in black (MERGED_DEL_2_33224, chr5:170129734-170131205, hg19). The inferred CNV region in the present study is represented in green.

Supplementary Figure 2

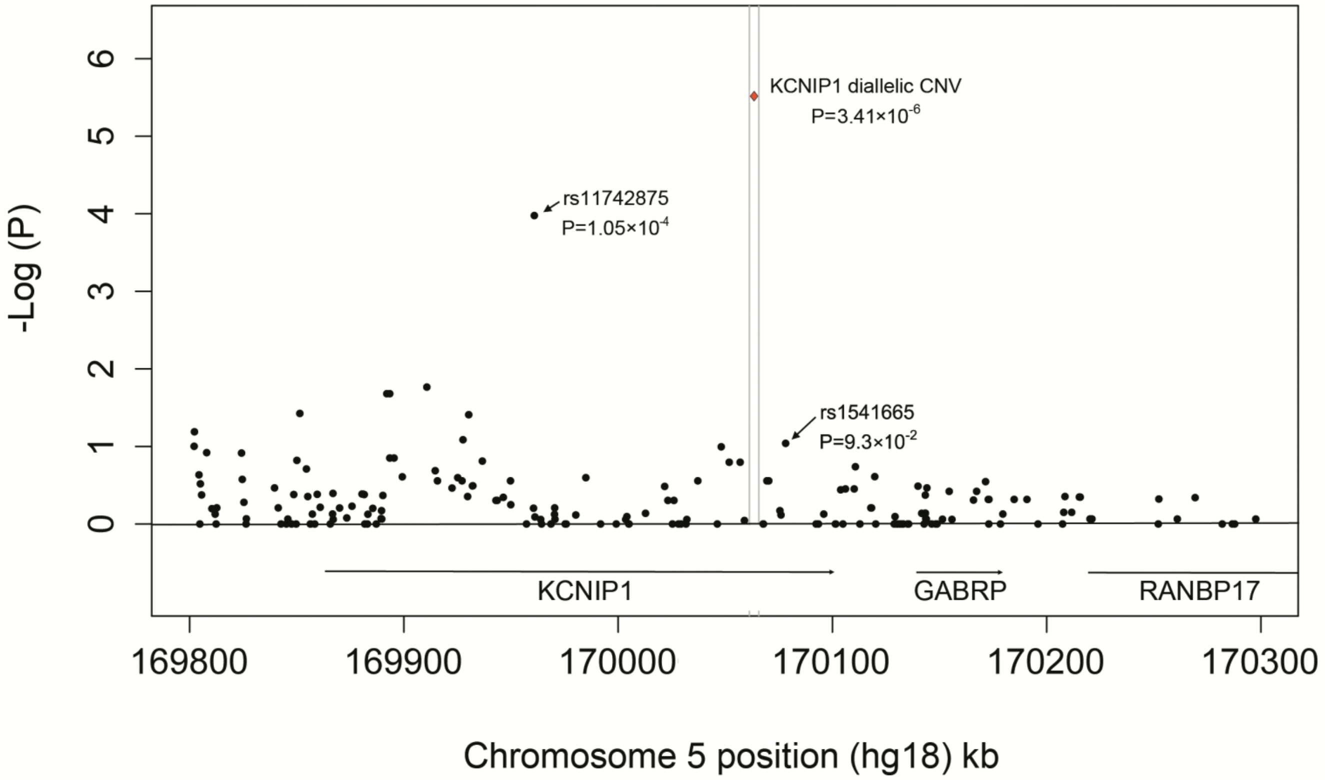

Supplementary Figure 2. Regional plots for the association of single nucleotide polymorphisms (SNPs) around the *KCNIP1* intron 1 CNV with atrial fibrillation (AF)**.** The regional plot for the association of SNPs selected from the humanOmni1-Quad BeadChip in the region flanking the *KCNIP1* intron 1 CNV with AF in the stage I sample (50 extreme AF cases and 50 NSR controls) is shown. For each SNP, the chromosomal location is shown on the x-axis and the significance level for association with AF is indicated by a $–\log_{10}P$ value on the y-axis. P-values are expressed as $–\log_{10}(P)$(y-axis) for every tested SNP ordered by chromosomal location (x-axis). Genomic position was determined using the NCBI database (NSCI Build 36). The two vertical lines define the predicted boundaries of the *KCNIP1* intron 1 CNV. Regional genes are also annotated below the panel. The *P*-value of the diallelic CNV is shown as a red diamond. The data were analyzed and the regional association plots were prepared with the R version 3.1.2 software (The R Project for Statistical Computing).

Supplementary Figure 3

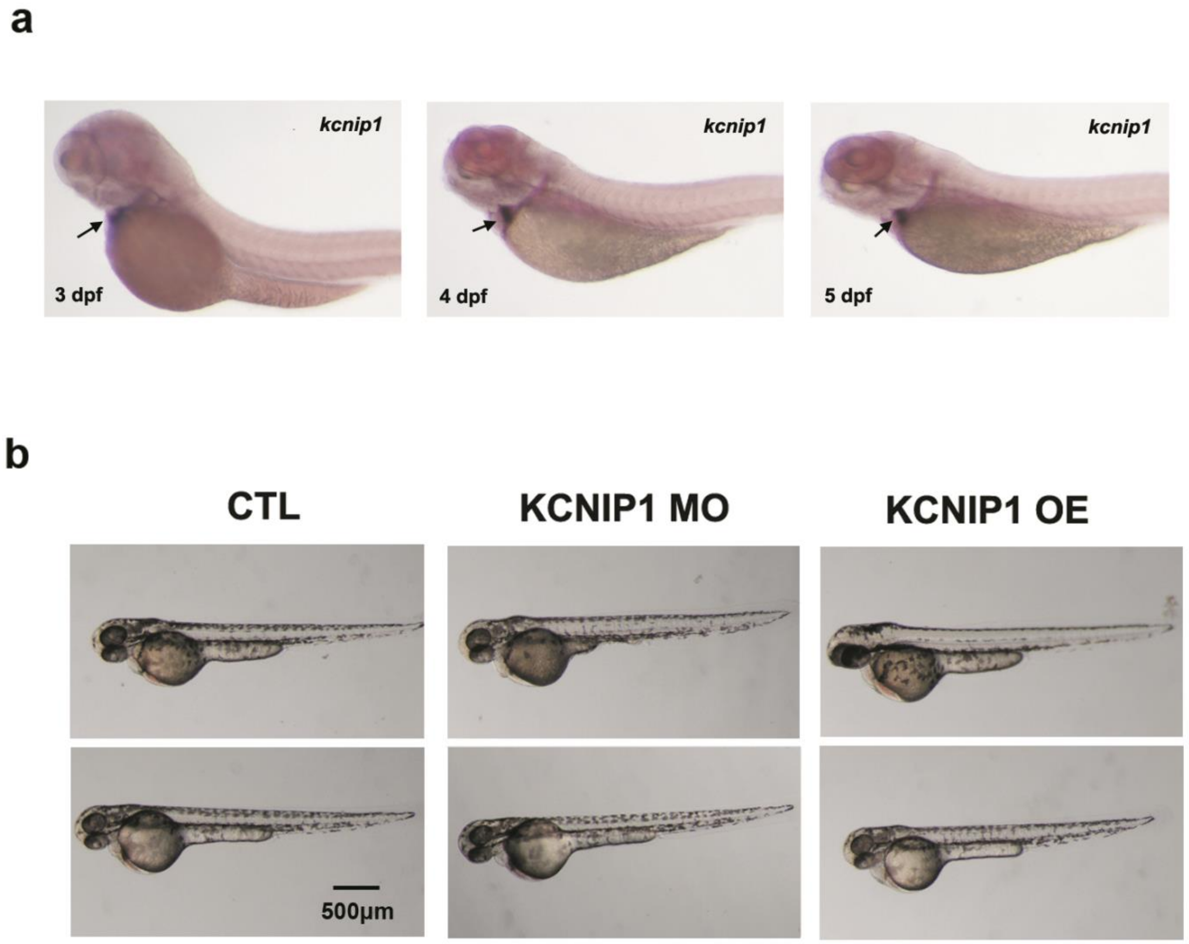

Supplementary Figure 3. Cardiac expression of *KCNIP1* and gross phenotypes of *KCNIP1*-knockdown, overexpression and control hearts. **(a)** Whole mount *in situ* hybridization of 3-dpf-, 4-dpf and 5-dpf-embryos shows expression (dark brown staining) of *KCNIP1* in the heart (arrows). dpf, days post-fertilization. **(b)** Bright-field micrographs of anesthetized 3 days post fertilization control embryos (CTL) and embryos injected with *KCNIP1* morpholinos (KCNIP1 MO) or mRNA (KCNIP1 OE). Scale bar, 500 μm.

Supplementary Figure 4. Full-length of the presented gels in Figure 1.

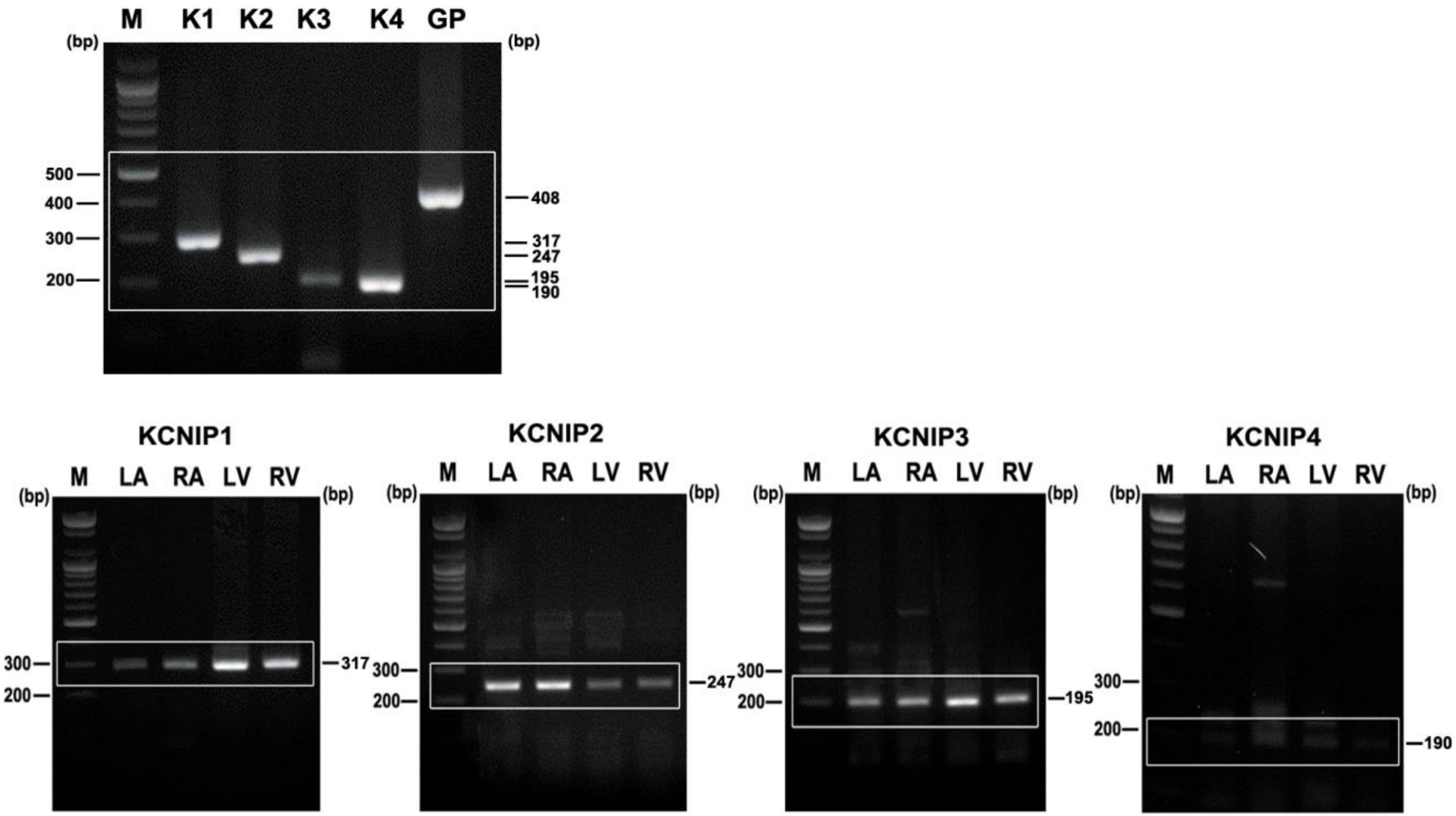

Supplementary FIgure 5. Full-length of the presented gels in Figure 2.

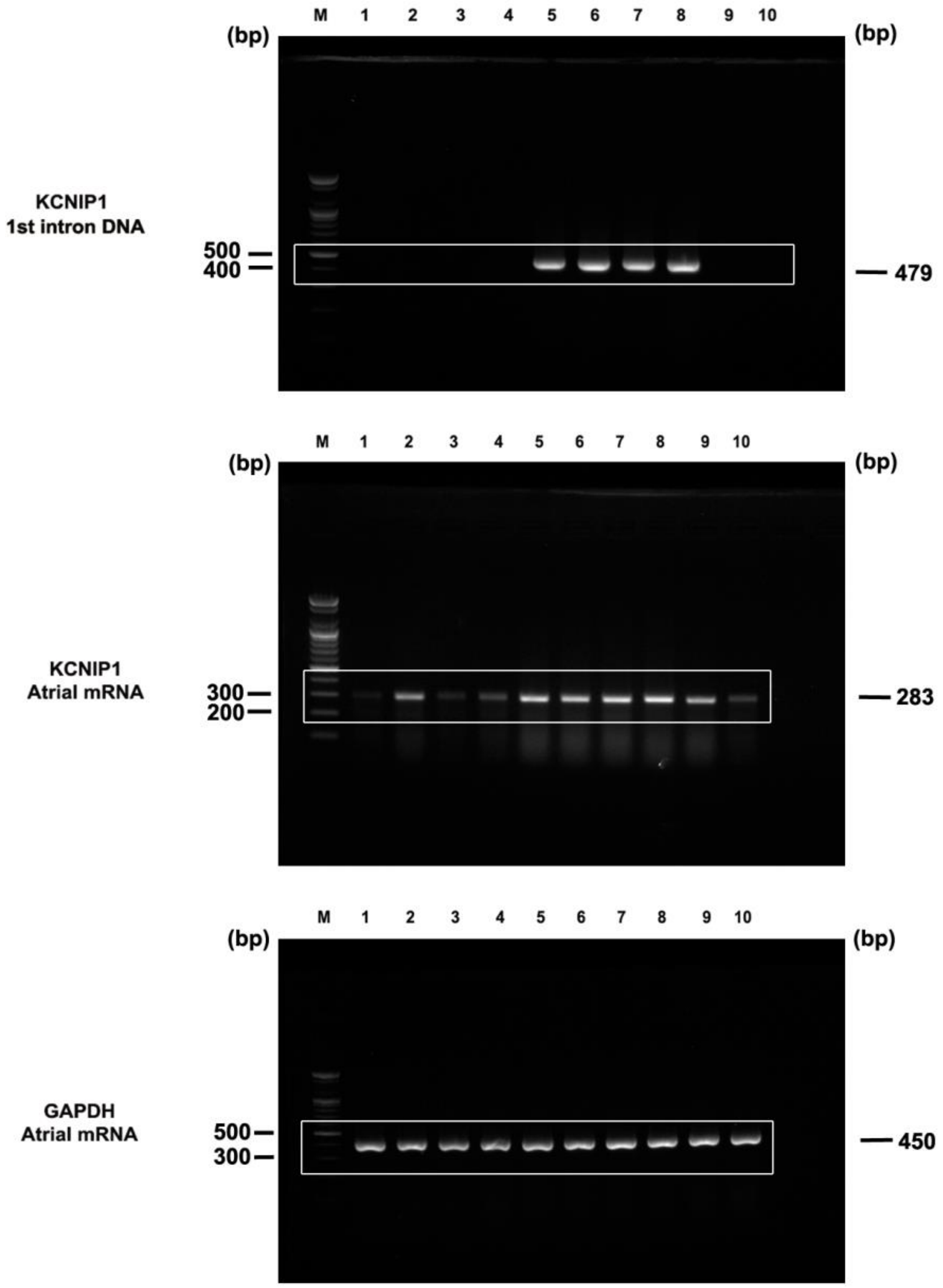

Supplementary Figure 6. Full-length of the presented blots in Figure 5.

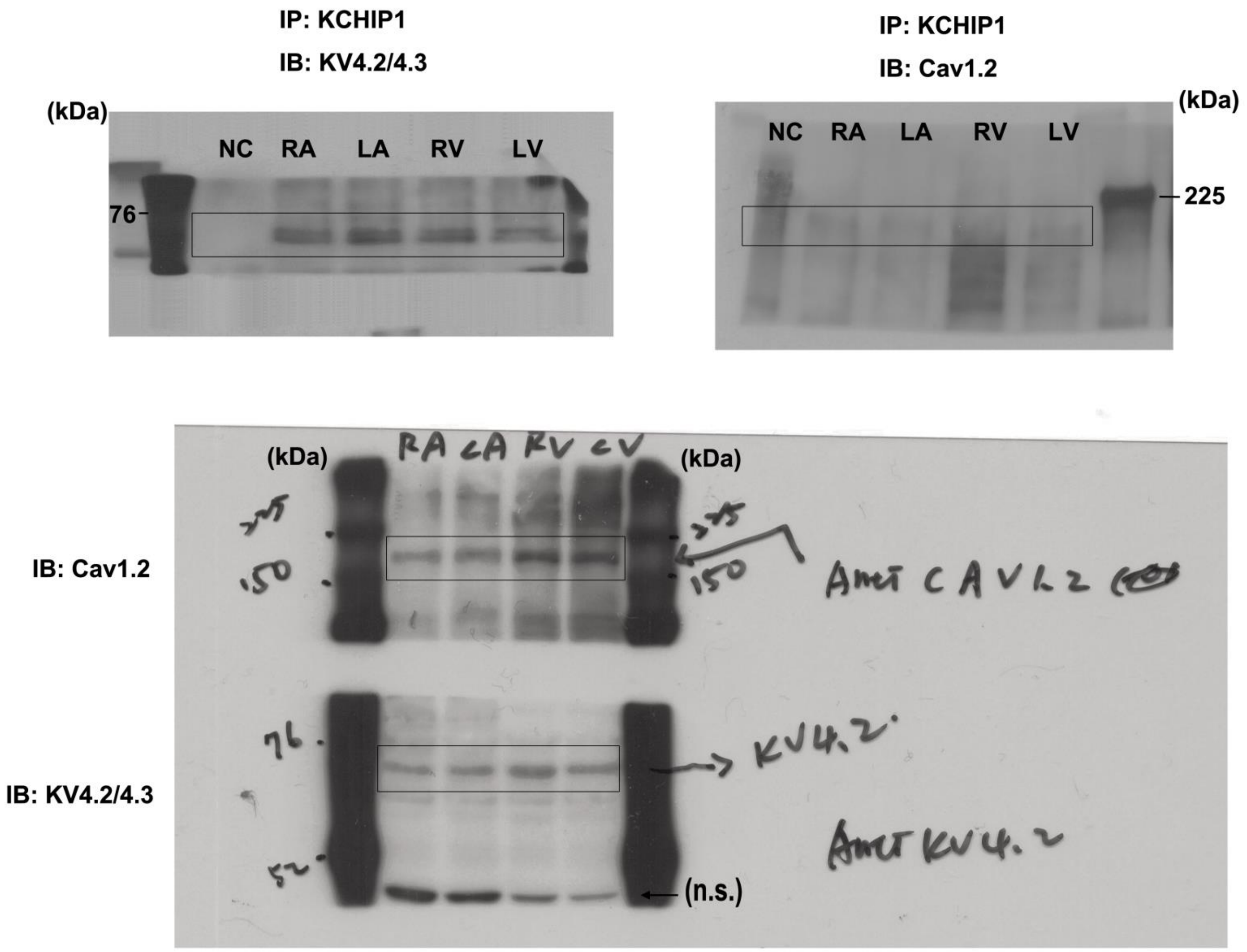

Supplementary Figure 7. Full-length of the blots presented in Figure 6.

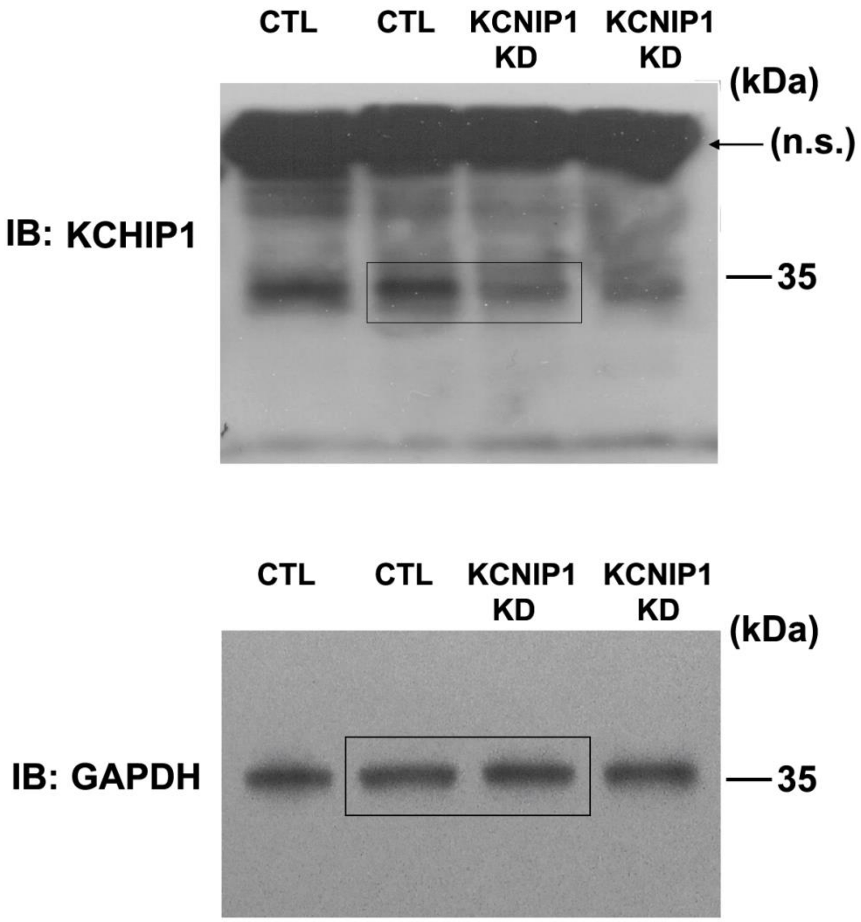

Supplementary Tables

Supplementary Table 1. Baseline Characteristics of Subjects in NTUH AF Registry

Variables	Stage I		Stage II		Stage III	
	AF (n=50)	Control (n=50)	AF (n=105)	Control (n=422)	AF (n=1021)	Control (n=2025)
Age, y	47.4±9.2	47.0±9.0	66.0±9.0	57.0±15.7	68.7±12.1	60.7±14.2
Gender (male/female), n	37/13	37/13	75/30	159/263	616/409	1109/916
Body height, cm	165±8	167±8	165±9	161±10	162±9	162±9
Body weight, kg	67±12	70±12	66±13	63±12	64±14	65±13
BMI, kg/m^2	24.2±3.2	25.9±3.4	24.6±5.4	24.0±3.9	24.4±4.6	24.8±4.0
CAD or MI (with/without)	—	—	11/94	—	258/763	402/1623
DM (with/without)	—	—	14/91	—	241/780	331/1694
HTN (with/without)	—	—	38/67	—	576/445	823/1202
VHD (with/without)	—	—	4/101	—	403/618	365/1660

AF, atrial fibrillation; BMI, body mass index; CAD, coronary artery disease; DM, diabetes mellitus; HTN, hypertension; MI, myocardial infarction; NTUH, National Taiwan University Hospital; VHD, valvular heart disease. Missing data were excluded from analysis.

Supplementary Table 2. Baseline characteristics of subjects in different geographic areas

Variables	AF (n=275)	Control (n=546)
Age, y	70.4±11.7	65.9±11.5
Gender (male/female), n	165/110	379/167
Body height, cm	161±9	161±9
Body weight, kg	63±15	66±13
BMI, kg/m^2	24.5±5.0	25.2±4.2
CAD or MI (with/without)	77/198	345/201
DM (with/without)	86/189	199/347
HTN (with/without)	159/116	412/134
VHD (with/without)	120/155	113/433

AF, atrial fibrillation; BMI, body mass index; CAD, coronary artery disease; DM, diabetes mellitus; HTN, hypertension; MI, myocardial infarction; VHD, valvular heart disease. Missing data were excluded from analysis.
